# Vacuum-assisted closure versus conventional dressing in necrotizing fasciitis: a systematic review and meta-analysis

**DOI:** 10.1186/s13018-023-03561-7

**Published:** 2023-02-04

**Authors:** Rongli Zhang, Yahui Zhang, Liyuan Hou, Chengyong Yan

**Affiliations:** Department of Orthopaedic Surgery, Third Hospital of Hebei Medical University, Shijiazhuang, China

**Keywords:** Necrotizing fasciitis, Vacuum-assisted closure, Conventional dressing, Meta-analysis

## Abstract

**Background:**

Necrotizing fasciitis is a rapid and severe soft tissue infection that targets subcutaneous fat tissue, muscle, and fascia. This study compares the clinical outcomes of vacuum-assisted closure (VAC) versus conventional dressing on necrotizing fasciitis.

**Methods:**

We systematically searched Embase, Cochrane, and PubMed for clinical trials (published between January 1, 1995 and September 30, 2021), which compared VAC with conventional dressing for necrotizing fasciitis. The mortality rate of necrotizing fasciitis was the primary outcome of this study. The number of debridements, the total length of hospital stay, and the complication rate were secondary outcomes. A random effects model assessed all pooled data.

**Results:**

A total of 230 identified studies and seven controlled clinical trials met the inclusion criteria and were included in this analysis (*n* = 249 participants). Compared to the conventional dressing, patients treated with VAC had a significantly lower mortality rate [OR = 0.27, 95% CI (0.09, 0.87)] (*P* = 0.03). Total length of hospital stays [MD = 8.46, 95% CI (− 0.53, 17.45)] (*P* = 0.07), number of debridements [MD = 0.86, 95% CI (− 0.58, 2.30)] (*P* = 0.24), and complication rate [OR = 0.64, 95% CI (0.07, 5.94)] (*P* = 0.69) were not significant. These results did not show significant differences between both groups treated with VAC or conventional treatment.

**Conclusion:**

VAC could significantly decrease the death rate compared to conventional dressing. No significant impacts were found on the number of debridements, the total length of hospital stay, and the complication rate in this study.

*Level of evidence* Level-III.

*Registration* Research Registry (reviewregistry1246).

**Supplementary Information:**

The online version contains supplementary material available at 10.1186/s13018-023-03561-7.

## Introduction

Necrotizing fasciitis (NF) is a rapid, severe, and life-threatening soft tissue infection that targets subcutaneous fat tissue, muscle, and fascia [1]. This disease usually occurs in the lower extremities, genitalia and perineum (Fournier’s gangrene, FG) [2]. NF has different names, including streptococcal gangrene, gas gangrene, suppurative fasciitis, Meleney’s gangrene, necrotizing erysipelas, and Fournier’s gangrene. Wilson first coined the term 'necrotizing fasciitis' to define both non-gas or gas-forming necrotizing infections of the subcutaneous tissue and fascia in 1951 [3].

Familiarity with the pathophysiological development of necrotizing fasciitis is essential for the early and rapid identification of clinical manifestations of disease. The main pathological changes of necrotizing fasciitis are in the superficial fascia. Bacteria colonize and multiply in the superficial fascia, secrete complex enzymes and toxins, and spread rapidly through the fascia [4]. Uncontrolled bacterial proliferation leading to vascular thrombotic microbial invasion with superficial fascial liquefaction and necrosis is the disease progression [5, 6]. Eventually, the skin becomes necrotic due to ischemia, with subcutaneous fat, dermis, and epidermis gangrene.

Surgically successful and timely diagnosis is essential in managing this rare and rapidly progressing disease. A high mortality rate of 12–20% has been reported, especially without early surgical intervention [7, 8]. After wound debridement and systemic antibiotics according to bacterial culture, a large open wound usually remains [9]. The wound is traditionally managed with the conventional dry or wet gauze technique before covering it with a skin graft, flap, or musculocutaneous flap. The morbidities associated with using conventional dressing techniques in handling exposed wounds could be extensive.

In 1997, Morykwas and Argenta first introduced the VAC based on a porcine model study [10]. After 20 years of development, this technology has been evaluated by several clinical and experimental studies and has been proven to promote the coverage of acute or chronic wounds [11–13]. VAC by pulling wound edges together to narrow the wound size, promoting granulation tissue formation on the wound bed for skin-grafting, promoting microcirculation, decreasing edema, and removing infectious tissues. To our knowledge, there were no meta-analysis and systematic review incorporating all these trials and comprehensively comparing VAC with conventional dressing techniques in treating NF patients. This meta-analysis study was conducted to compare these two treatments and provide clinically referable evidence about clinical outcomes.

## Materials and methods

This recent systematic review and meta-analysis were conducted according to the Cochrane handbook guideline for systematic reviews of interventions [14].

### Search strategy and selection criteria

We searched for all relevant trials published between January 1, 1995 and September 30, 2021, on Embase, PubMed, and Cochrane. The following research strategy, which combined with several MeSH terms, was used in each database: ("necrotizing fasciitis" OR "necrotizing fascitides" OR "necrotizing fasciitides" OR "necrotizing fasciitis") AND ("vacuum-assisted closure" OR "negative pressure" OR "subatmospheric pressure" OR "suction dressing" OR "topical negative pressure" OR "VAC" OR "vacuum therapy") (Additional file 1). We conducted a manual search based on the references of important articles published in English.

### Study selection and data extraction

Two independent investigators (R.L.Z. and Y.H.Z.) reviewed the titles and abstracts of all searched studies, and the studies that met the conditions were downloaded for full-text reading. Disagreement between the two reviewers was resolved by consensus. The senior author made the final decision if a consensus could not be reached.

The inclusion criteria were cohort studies that compared VAC and conventional dressing therapy in NF. Exclusion criteria included the following: (1) the study reported a case or case series; (2) no English-language articles; (3) the study without outcome measures; (4) letter, commentary, editorial or systematic review.

The data from each selected study were extracted: first author, publication year, study type, the total number of participants, and age. The primary outcome was the mortality rate in both treatment groups. Secondary outcomes were the number of debridements, the total length of hospital stay, and the rate of complications. The studies in which the mean and standard deviation were not supplied were estimated statistically using the relevant data [15].

### Quality assessment

The quality of all enrolled studies was assessed according to the classic Newcastle Ottawa Scale scores (NOSs) [16]. NOSs consist of eight items with three subscales and range from 0 to 9 points. A study with a score of 7–10 has high quality, 4–6 moderate quality, and 0–3 poor quality.

### Statistical analysis

Review Manager 5.4 (The Nordic Cochrane Center, Copenhagen, The Cochrane Collaboration) was applied for statistical analysis. MD (mean difference) was used to analyze continuous variables, and OR (odds ratio) was used to present the dichotomous variables. A random effect model pooled all extracted data if *I*^2^ ≥ 50% between included studies was statistically significant. Otherwise, the fixed-effect model was selected. All variables were reported with 95% CI (confidence interval). Statistical heterogeneity between studies was calculated with chi-square and *I*^2^ tests. *I*^2^ values between 0 and 30% indicated homogeneity, 30–60% indicated moderated heterogeneity, and values above 60% indicated substantial heterogeneity. A two-sided *P* value < 0.05 was considered statistically significant. Funnel plot analysis was used to reflect publication bias. According to Egger et al. [17], the ability of funnel plots to detect this bias is limited when the number of included studies is small. Consequently, we ensure to provide the funnel plot as a supplementary file.

## Results

### Search results

**Fig. 1:**
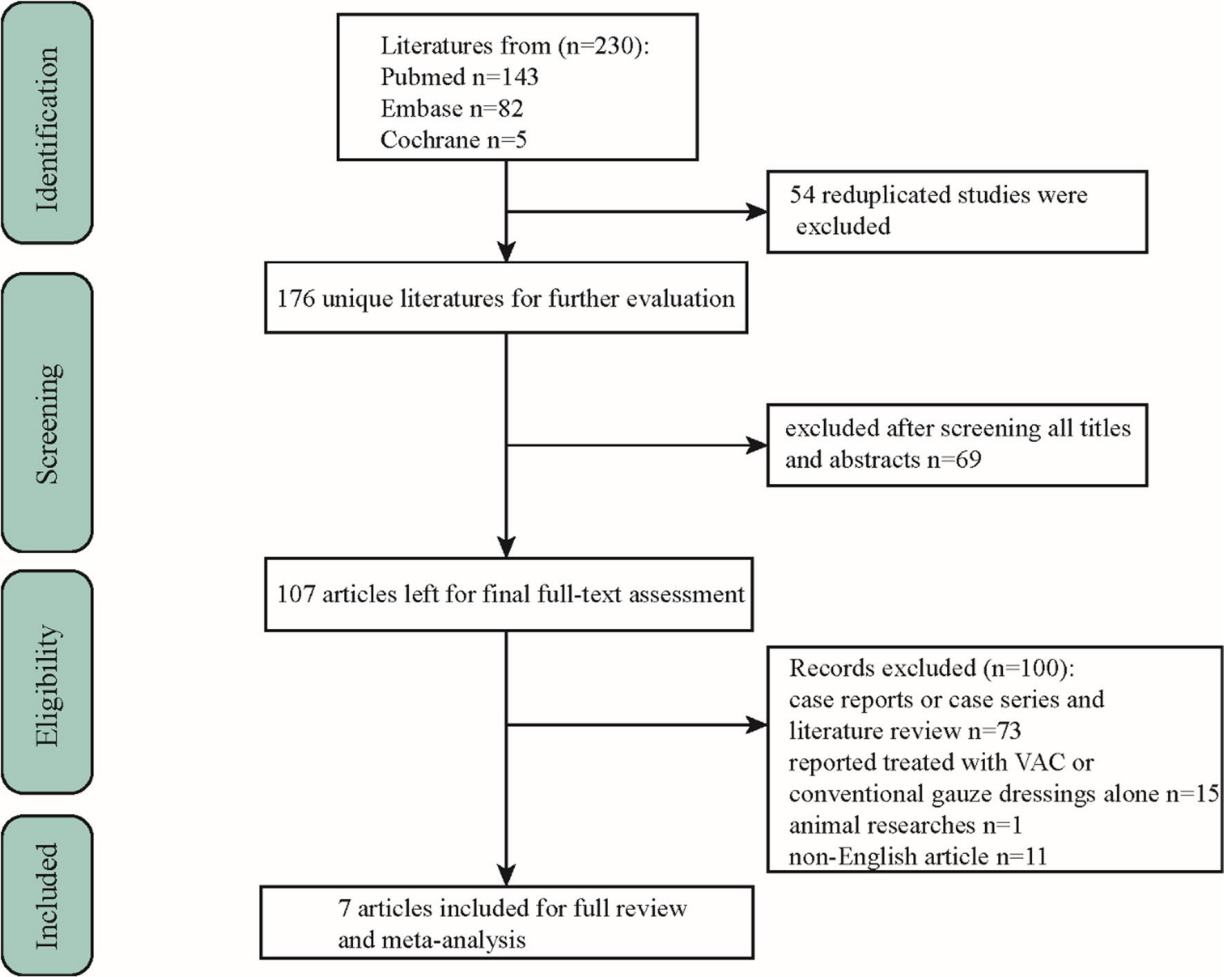
Flow diagram of studies included in the systematic review

Two authors (L.Y.H. and C.Y.Y.) retrieved 230 literature from these three databases. Among them, 54 studies were repeated and excluded. Then, by reviewing the titles and abstracts of the remaining research, 69 studies were eliminated according to inclusion and exclusion criteria, and 100 were eliminated after reviewing the whole texts for these reasons: case reports or case series and literature review, cohort studies separately reported treated with VAC or conventional gauze dressings, animal studies, and non-English article. Finally, seven cohort studies [18–24], including 249 NF cases, were eligible for literature review, data extraction, and meta-analysis. Figure 1 shows a summary of the trial screening process.

### Characteristics of the included studies

Table 1 shows a summary of all enrolled studies. All these seven research studies were published between 2006 and 2021. Ultimately, seven cohort studies involving 249 NF patients were included in this review. The number of patients in these seven research studies ranged from 11 to 92. All these studies compared VAC with conventional dressing treatment and reported its efficacy. Outcomes of mortality rate, number of debridements, the total length of hospital stay, and complication rate were extracted for pooling the results. Since a random effect model was adopted, the publication bias risk for the main outcome (mortality rate) was presented by funnel plot and illustrated in Additional file 2: Fig S1. The NOS score was "high quality (ranging from 7 to 9 points)" in all included studies (Table 1).

**Table 1:** The main characteristics of the included studies

Year, First author	Study design	VAC VS conventional dressing					Location	NOS
		Number (VAC vs. conventional dressing)	Age	CRP	Laboratory risk indicator for necrotizing fasciitis (LRINEC)	Management of the wound		
Iacovelli et al. [19]	CS	33 vs. 59	64.25 (54–75), 69.5 (58,82)	165.7 (45.0–309), 55.45 (9.6–223.8)	–	VAC: 75–125 mmHg negative pressure, with 5 min of suction followed by 2 min rest Conventional dressing: Hydrogen peroxide and povidone/iodine solution were used mainly to soak dressings. The wound dressing was changed daily	Perineum or perianal region (Fournier’s Gangrene)	9
Gul et al. [21]	CS	12 vs. 10	55.50 ± 10.39, 58.10 ± 14.39	24.78 ± 8.81, 16.78 ± 12.25	–	VAC: debridement + VAC Conventional dressing: debridement only	Perineum or perianal region (Fournier’s Gangrene)	8
Zhang et al. [18]	CS	10 vs. 1	60 (45–71)	–	10.27 ± 1.61, 8	VAC: negative pressure ranging from − 120 to − 125 mmHg, changed every 3–7 days	Perineum or perianal region (Fournier’s Gangrene)	7
Mustafa et al. [22]	CS	8 vs. 3	55.00 ± 7.75, 63.00 ± 14.73	27.56 ± 8.53, 80.33 ± 80.75	6.88 ± 1.36, 6.67 ± 2.52	VAC: intermittent therapy at − 100 mmHg, cycling at 5 min on and 2 min off	Head and neck	7
Yanaral et al. [24]	CS	23 vs. 31	61.6 ± 7.6, 55.8 ± 14.9	–	–	VAC: Initially, the pressure is set at 50 mm Hg and increased to a maximum of 125 mm Hg. VAC dressings were changed every 48–72 h. Conventional dressing: patients' wounds were covered with conventional antiseptic dressings. Wound dressings were changed twice a day	Perineum or perianal region (Fournier’s Gangrene)	7
Czymek et al. [23]	CS	19 vs. 16	57.2, 58.2	–	–	VAC: maintained − 125 mmHg, removed every 48–72 h. Conventional dressing: treated with gauze dressing soaked with physiological saline solution, changed three to six times a day	Perineum or perianal region (Fournier’s Gangrene)	9
Huang et al. [20]	CS	12 vs. 12	57.75 (35–78), 62.58 (36–85)	–	–	VAC: maintained − 125 mmHg, removed every 48–72 h Conventional dressing: treated with gauze dressing soaked with physiological saline solution, changed three to six times a day	Extremity	8

*CS*, cohort study; *CRP*, C-reactive protein; *NOS*, Newcastle Ottawa Scale scores; *VAC*, vacuum-assisted closure

## Meta-analysis of clinical outcomes

### Mortality rate

**Fig. 2:**
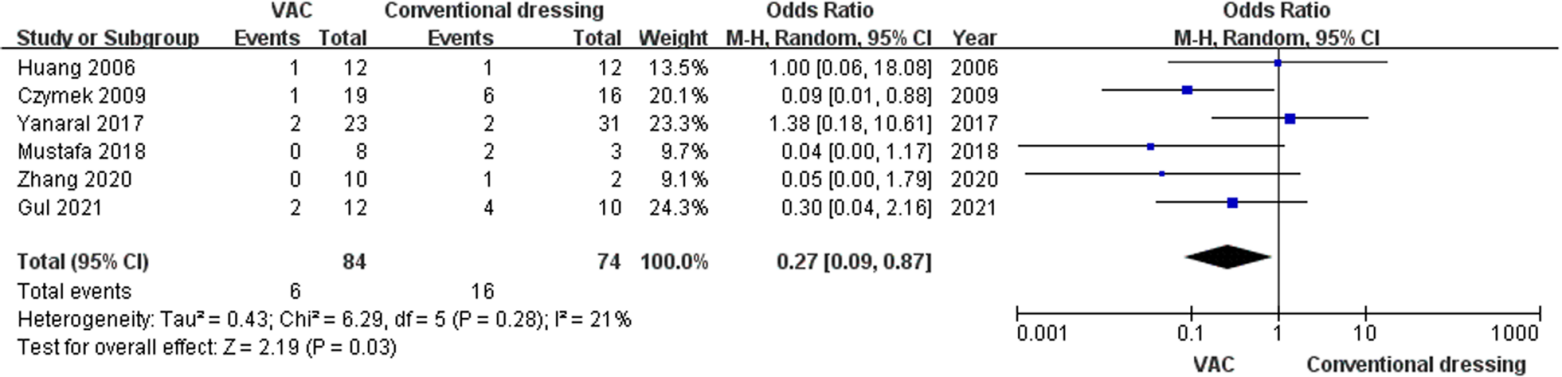
Forest plot of the odds ratio of mortality rate between VAC and conventional dressing

Six studies involving 151 patients reported the mortality rate of NF after being treated with VAC or conventional dressing. Under the random effects model (Fig. 2), the mortality rate of NF patients in VAC group was lower than in the conventional dressing group (OR, 0.27; 95% CI, 0.09–0.87), and it has statistical significance (*P* = 0.03). No statistically significant heterogeneity was observed between these VAC and conventional dressing groups (*P* = 0.28, *I*^2^ = 21%).

### Total length of hospital stay

**Fig. 3:**
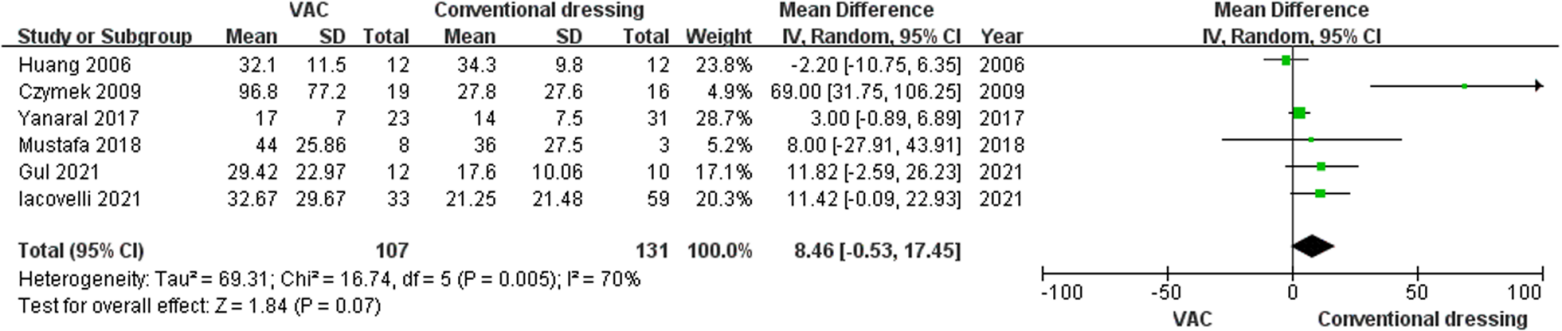
Forest plot of the mean difference of total length of hospital stay between VAC and conventional dressing

Six studies (238 patients) compared the total hospital stay of NF after being treated with VAC or conventional dressing. Pooled results revealed no statistical difference in the total length of hospital stay between these two groups (MD, 8.46; 95% CI, − 0.53–17.45; *P* = 0.07). There was significant heterogeneity between these studies (*P* = 0.01, *I*^2^ = 70%), and the random effects model was applied for meta-analysis (Fig. 3).

A sensitivity analysis was conducted to evaluate the influence of every single study on the meta-analysis outcome by excluding studies one by one. When eliminating the study performed by Czymek [23] and recalculating the other research studies, the heterogeneity of the total length of hospital stay was decreased from 70 to 19%.

### Number of debridements

**Fig. 4:**
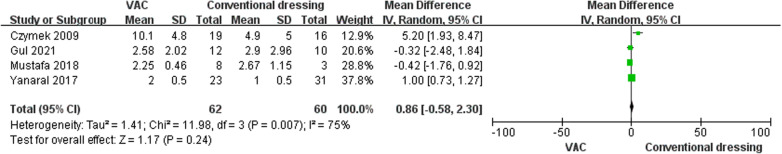
Forest plot of the mean difference in the number of debridements between VAC and conventional dressing

Four studies (62 in VAC group and 60 in the conventional dressing group) compared the number of debridements of NF after being treated by VAC or conventional dressing. The pooled MD of number of debridements between these two groups was MD = 0.86 (95% CI, − 0.58–2.30), which was not statistically significant (*P* = 0.24). The random effects model was used in the meta-analysis, and a significant heterogeneity (*I*^2^ = 75%) was found between these studies (*P* = 0.01) (Fig. 4).

When performing the sensitivity analysis, after excluding the studies one by one, it was found that the heterogeneity could not be reduced below 60%. Heterogeneity was lowest at 63% when the study by Czymek [23] was excluded.

### Complication rate

**Fig. 5:**
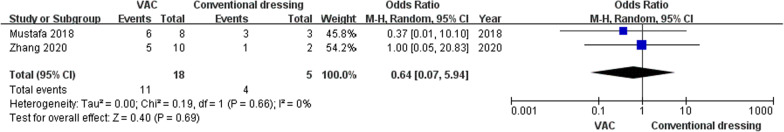
Forest plot of the odds ratio of complication rate between VAC and conventional dressing

Two studies reported the rate of NF complication after being treated with VAC or conventional dressing. The pooled OR for the complication rate was 0.64 between these two groups, which showed no difference (95% CI, 0.07–5.94, *P* = 0.69). No heterogeneity was revealed between these two treatments (*P* = 0.66, *I*^2^ = 0%), and a random effects model was applied (Fig. 5).

## Discussion

This systematic review and meta-analysis included seven cohort studies (249 cases). All relevant observed indicators from the seven selected studies were extracted and pooled. After several analyses, the most important finding of this meta-analysis study is that using VAC could decrease the mortality rate significantly compared to conventional dressing for treating NF patients. However, there was no evidence that VAC could reduce the length of hospital stay, number of debridements, and complication rate.

Treating NF patients is based on complete debridement and antibiotherapy. Recurrent surgical debridement should be performed to remove necrotic soft tissue and ensure sufficient drainage. However, treating the wound that occurs secondary to debridement in patients with NF can be difficult. The complications associated with conventional dressing techniques in managing the residual wound could be diverse. The use of vacuum-assisted closure, such as VAC, for NF infection, has attracted much attention recently [25]. When used in treating NF, it could reduce daily gauze changes and toxin absorbance, resulting in less pain and decreasing narcotic use. Moreover, VAC is useful to preserve residual subcutaneous soft tissue and promote the formation of a better wound bed, which is essential for later reconstructive surgery [26–29].

This study is the first literature review and meta-analysis to assess VAC and conventional dressing in treating NF patients. Bacteremia and sepsis secondary to necrotizing fasciitis will lead to multiple organ failure and high mortality. The mortality rate of NF is an important parameter in assessing the therapeutic outcome of NF. In this present study, the main outcome was the mortality rate. Among the seven included studies, six documented the mortality rate of NF [18, 20–24]. The combined odds ratio (OR) showed that VAC could reduce mortality by 27% compared to conventional dressing. Among these seven studies, five were NF of the perineal region (Fournier's Gangrene) [18, 19, 21, 23, 24], one was located head and neck [22], and one was located extremities [20]. The mortality of conventional dressing group in head and neck necrotizing fasciitis (HNNF) has the highest mortality rate (66.7%). Consequently, the perineal area (37.5–50%). NF usually appears in the lower extremities and abdominal wall. Previous literature has reported a mortality rate of HNNF as high as 70% [30] because of severe sepsis, acute renal failure, necrotizing mediastinitis or multi-organ failure. The main treatment for Fournier's Gangrene is the complete debridement of necrotic area and administration of empirical broad-spectrum antibiotics to prevent disease progression. However, it is difficult to keep the perineal region covered with a conventional dressing and clean; VAC might be a treatment of choice.

Seven trials in the present meta-analysis reported no significant differences in total hospital stay [19–22]. The length of hospital stay will be lengthened by large tissue defects with exposure of the wound or bacteremic complications up to the axillary and perineal regions. Czymek et al. claimed that the mean length of hospital stay of NF was 96.8 days in the VAC group and 27.8 days in the conventional dressing group [23]. The reason for that was the surgeon did not apply VAC immediately after the first-time debridement but after the second or the third surgery. This may also be one of the reasons why sensitivity analyzes showed increased heterogeneity due to the presence of the study. Czymek et al. concluded that although VAC group stayed much longer than conventional group, VAC could provide a cleaner wound without exudate, even in deep or problematic wounds.

The key to NF treatment is based on prompt and entire debridement. Surgery aims to remove all necrotic tissues to prevent the progression of infection and minimize the general reaction of NF patients. Four studies in the present meta-analysis revealed no significant differences in the number of debridements [21–24], and three of the four articles reported on Fournier's Gangrene treatment [21, 23, 24]. Yanaral et al. [24] claimed that the time and degree of first debridement are the most important risk factors affecting the mortality rate. Chawla et al. documented that an average of 3.5 debridements was efficient in treating NF [31]. After the first surgical debridement, wound management and adequate nutrition for the patient are essential. Subsequent debridement was performed if necessary.

Complications, including sepsis, respiratory failure, and multiple organ failure, usually occur in NF patients. Corresponding treatment methods, such as blood filtration, dialysis, and ventilator therapy, were adopted for these patients. Of seven included studies, only two had documented the rate of NF complication [18, 22]. The merged odds ratio (OR) showed no significant between VAC and conventional dressing. High-risk factors for NF include advanced age, diabetes mellitus, hypertension, alcohol use, and peripheral vascular or nerve disease. There are several scoring systems, such as neutrophil lymphocyte rate (NLR), laboratory risk indicator for necrotizing fasciitis (LRINEC), and Fournier's Gangrene Severity Index (FGSI) [32]. A previous study reported that an FGSI score greater than nine points was related to a higher mortality rate [33]. In 2004, Wong et al. reported LRINEC score as a predictive index capable of predicting early NF patients [34], a score of no less than 8 is strongly diagnostic of Fournier's Gangrene. In this present meta-analysis study, Zhang et al. [18] and Mustafa et al. [22] used LRINEC score to assess the risk of necrotizing fasciitis. In contrast, the studies by Iacovelli et al. [19] and Gul et al. [21] used FGSI score. Future research must prove which score is clinically meaningful or propose a more widely accepted scoring method in clinical work.

This present study had some limitations. First, NF is a rare and rapid subcutaneous soft tissue infection disease. Most searched studies in this study were case or case series reports. Unfortunately, high-quality RCTs were impossible to find. Second, the seven selected studies included patients with different locations of NF. Inconsistent baseline and distribution of NF may have been an influencing factor of clinical heterogeneity.

## Conclusions

This present meta-analysis study displayed that VAC could reduce the mortality rate of necrotizing fasciitis patients. However, no significant differences were found between VAC and conventional dressing groups regarding the number of debridements, the total length of hospital stay, and the complication rate. More VAC versus conventional dressing trials are required from a randomized design perspective to provide a better evaluation of all outcome measures.

## Supplementary Information

**Additional file 1**. MeSH terms and research strategy used in the search process.

**Additional file 2**. **Fig. S1**. Publication bias funnel plot for mortality rate

## Data Availability

All data generated or analyzed during this study are included in this published article.

## Abbreviations

VAC: Vacuum-assisted closure
NLR: Neutrophil lymphocyte rate
LRINEC: Laboratory risk indicator for necrotizing fasciitis
FGSI: Fournier’s gangrene severity index
OR: Odds ratio
HNNF: Head and neck necrotizing fasciitis
MD: Mean difference
NF: Necrotizing fasciitis
FG: Fournier’s gangrene
CI: Confidence interval
