# Supplementary material for: Vacuum-assisted closure versus conventional dressing in necrotizing fasciitis: a systematic review and meta-analysis
Source: J Orthop Surg Res. 2023 Feb 4;18:85. doi: 10.1186/s13018-023-03561-7 (PMC9898985; doi:10.1186/s13018-023-03561-7)
Supplement: Supplementary file 1 — Additional file 1. MeSH terms and research strategy used in the search process. [file 13018_2023_3561_MOESM1_ESM.docx]

The following research strategy which combined with several MeSH terms were used in each database (Embase, PubMed and Cochrane): (“necrotizing fasciitis” OR “necrotizing fascitides” OR “necrotizing fasciitides” OR “necrotizing fasciitis”) AND (“vacuum assisted closure” OR “negative pressure” OR “subatmospheric pressure” OR “suction dressing” OR “topical negative pressure” OR “VAC” OR “vacuum therapy”). The search time interval of these three databases is set from Jan 1, 1995 to September 30, 2021.
