# Supplementary figures and images for: Vacuum-assisted closure versus conventional dressing in necrotizing fasciitis: a systematic review and meta-analysis
Source: J Orthop Surg Res. 2023 Feb 4;18:85. doi: 10.1186/s13018-023-03561-7 (PMC9898985; doi:10.1186/s13018-023-03561-7)

**
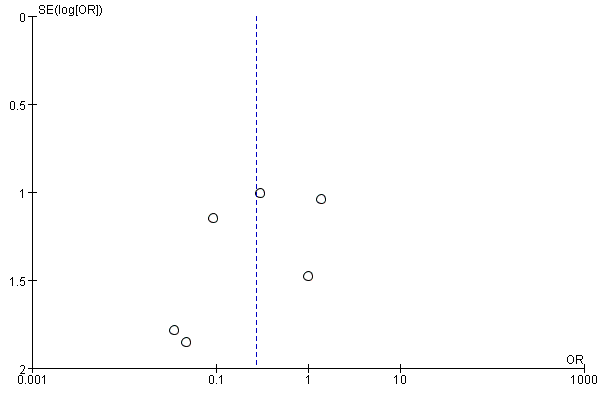
**

**Fig.** Publication bias funnel plot for mortality rate.

Supplement: Supplementary file 2 — Additional file 2. Fig. S1. Publication bias funnel plot for mortality rate [file 13018_2023_3561_MOESM2_ESM.docx]
